# Does salary affect the choice of residency in non-university teaching hospitals? A panel analysis of Japan Residency Matching Programme data

**DOI:** 10.1186/1478-4491-11-12

**Published:** 2013-03-08

**Authors:** Taiji Enari, Hideki Hashimoto

**Affiliations:** 1 Department of Health Economics and Epidemiology Research, School of Public Health, The University of Tokyo, 7-3-1 Hongo, Bunkyo-ku, Tokyo 113-0033, Japan

**Keywords:** Residency programme, Economic incentive, Matching programme, Japan

## Abstract

**Background:**

Previous studies have investigated factors that are influential on the choice of training hospitals among residency physicians, but the effect of salary was not conclusive. In this study, we aimed to examine whether a higher salary attracted more residents to non-university hospitals participating in the Japanese Residency Matching Programme.

**Methods:**

Data on 475 hospitals/programmes between 2006 and 2009 were available for analysis. We first conducted an ordinary least squares regression analysis on the ratio of the number of applicants to the residency programme quota as an index of resident’s choice, for comparison with previous studies. We further performed panel data analysis to better control for unobserved heterogeneity across hospitals, which could be confounded by the amount of salary. We also performed stratified analysis by the population size of the hospital location.

**Results:**

In ordinary least squares regression, salary showed a positive, but not significant association, with the ratio of the number of applicants to the programme quota, while the results of a fixed effect model exhibited a positive and significant effect of salary (ε= 0.4995, *P* = 0.015) on the ratio. Analysis stratified by city size showed that the elasticity of salary was comparable (ε= 1.9089, *P* = 0.016 in large cities versus ε= 1.9185, *P* = 0.008 in small cities), while that of the number of teaching physicians was larger in large cities (ε= 1.9857, *P* = 0.009) compared with that in small cities (ε= 1.6253, *P* = 0.033). The number of teaching physicians had a significant and negative effect modification on salary, implying an antagonistic effect between these two attributes (ε= −1.5223, *P* = 0.038).

**Conclusions:**

Our results indicate that the amount of salary influences the choice of training hospitals among medical graduates who choose non-university settings. Use of a monetary reward in a residency programme could be a feasible tactic for hospitals to attract residents.

## Background

The mal-distribution of physicians is a common problem not only in developed countries, but also in middle income and developing countries [1-9]. Possible political solutions to expand physicians to underserved areas are much discussed, such as increasing the supply of physicians, reforming career selection and professional training system, compulsory duties, and preparing incentives to serve in underserved areas [10]. Several studies have shown that increasing the supply of physicians falls short of a solution to the problem, but rather exacerbates the unequal distribution of physicians [2,10-12]. Instead, financial incentives have been regarded as more effective strategies to attract physicians in underserved areas, though they may not solve the whole issue of mal-distribution of the physicians [6,10,13-15].

Many studies have demonstrated that monetary incentives are influential on a physician’s career choice of subspecialties, such as paediatrics [16], emergency medicine [17], internal medicine [18,19], surgery [20], primary care [21,22], and being a general practitioner [23]. A previous study in the USA showed that the effect of concerns on lifestyle and income has increased over time in the career choice among new medical school graduates [24]. In Japan, Kawamura [25] performed one of the first studies to empirically assess influential factors of residency choice among new graduates. Using a multivariable regression model, this previous study concluded that salary was a significantly influential factor among those who chose university hospitals, while non-economic factors, such as the number of supervising doctors, were influential among those who chose non-university hospitals.

However, it is premature to conclude from Kawamura’s study [25] that the effect of payment conditions for residency choice is limited to those residents in university hospitals. In fact, according to Kawamura, the salary for 2 years in university hospitals was 6 478 000 yen ($80 975 at the exchange rate of 1 USD = 80 JPY) on average, compared with 9 115 000 yen ($113 938) in non-university hospitals [25]. Lower salaries in university hospitals were often attributed to authoritarian influence by clinical departments in medical schools on young trainees’ future career paths in affiliated hospitals [26]. Therefore, it is possible that a young graduate’s choice of non-university hospitals in his/her early career is already affected by incentives other than authoritative reasons, including economic incentives related to annual salary.

According to a questionnaire survey conducted by the Ministry of Health, Labour, and Welfare (MHLW), 27.1% of residents who chose non-university hospitals answered that salary and work environment affected their choice of training hospitals [27]. Nomura *et al*. also conducted a questionnaire survey among second-year residents, and reported that those who chose non-university hospitals were more satisfied with a higher income [28].

A wide variation in the annual salary, even among non-university hospitals, has been reported, with five- to six-fold difference between the top and bottom salaries [29]. The amount of salary is determined endogenously based on conditions in hospitals, such as location, university affiliation, teaching status, and size. Therefore, the effect of income on specialty choice might have been underestimated in previous studies because the amount of salary was treated as an exogenous variable. Hay showed that a significant effect of income on the choice of specialty was observed only when the simultaneously determined supply and demand of the specialty were counted by using a two-staged logit-ordinary least squares (OLS) estimator [30].

The objective of the current study was to identify whether the improved payment conditions affected a medical graduate’s choice of residency programme in Japan. We re-investigated the effect of annual salary on the hospital choice of medical graduates using a different approach. First, we limited our analysis to non-university hospitals because the distribution of salaries is different between university and non-university hospitals. In fact, salaries paid in university hospitals are homogenous across institutes because of regulatory reasons due to the governmental subsidy. The salaries in university hospitals have also been almost constant over time since the introduction of the new residency programme as is described shortly [31]. Furthermore, the choice of university hospitals is supposedly influenced by a distinctive set of individual preferences, such as a preference for an academic career path and for home location [27]. Because data on individual preferences were not available, exclusion of university hospitals from our study sample was necessary to discriminate the effect of salary and distinctive sets of individual preferences. Additionally, because university hospitals often change their quota for residents according to the expected number of applicants, the ratio of the number of applicants to the quota, which was our target variable, is endogenously determined, and does not necessarily reflect residents’ preferences in the case of university hospitals.

Second, we introduced panel data analysis to control for unobserved heterogeneity across hospitals that could be related to a hospital’s decision on the amount of salary. Therefore, we hoped to overcome any bias caused by an endogenously determined annual salary, and by controlling this, we aimed to determine the actual effect of salary on a new graduate’s choice of residency programme [32].

## Methods

### New Residency Matching Programme in Japan

In 2004, the Japanese government introduced a new Postgraduate Medical Education Programme (PGME) to improve physicians’ skills in general medicine. Before 2004, medical graduates tended to seek residency positions in university hospitals affiliated with their own graduate medical schools, where the residency programme was not standardized and was often limited to subspecialty training [33]. Since the introduction of PGME, all medical graduates are obliged to take the 2-year standardized residency programme. In addition, a nationwide computer-based residency matching system called the Japan Residency Matching Programme (JRMP) started to offer medical graduates a broader range of choices, including community hospitals with new residency programmes [34,35]. Because the number of resident positions exceeded the number of annual medical graduates, hospitals competed with each other to attract new medical graduates through appealing to the excellence of the training programme, resulting in a considerable shift of residents from university to non-university hospitals [28,36].

Another change since this reform was the payment conditions of residents. The annual salary of residents was raised, on average, up to 4.36 million yen ($ 54 500) after the reform in 2007 compared with 2.65 million yen ($ 33 100) before the reform in 2003 [27]. We took this reform as a natural laboratory to test whether salary could be an incentive to attract residents.

### Data collection

We obtained the number of applicants and the quota of each programme from publicly available data in the homepage of the JRMP [37]. The JRMP issues the interim and final annual reports. We used the numbers in the interim report because they better reflected the original preference of medical graduates before adjustment to the final decision. Because of data availability, we used data in 2006 and 2009 to obtain as large a sample size as possible for a balanced panel.

We obtained information on hospital characteristics from the Guidebook of Residency Programme Hospitals, which is annually published on the web site by the Foundation for Promotion of Medical Training [38,39]. This Guidebook contains information from approximately 800 teaching hospitals, including university hospitals, such as location, hospital size, the annual number of outpatients, inpatients, and emergency services, the number of medical staff and supervisor physicians, the contents of training programmes, and salary and other employment conditions for residents.

In 2006 and 2009, 1049 programmes of 944 non-university hospitals, and 1051 programmes of 940 non-university hospitals, respectively, participated in the JRMP. Among these, 796 programmes from 705 hospitals in 2006 and 793 programmes from 682 hospitals in 2009 provided information for the Guidebook. Because programmes were renamed, scrapped, or built over time, we manually examined the programme name, contents, and the size of the quota to match identical programmes between the 2 years. Some hospitals provided multiple programmes, although these programmes likely shared institution-specific conditions, such as hospital size and salary. Therefore, we selected one of the largest programmes per hospital. Otherwise, we used summation numbers across programmes to obtain the applicant/quota ratio of the whole institute. We were able to match 475 pairs of programmes for further analysis. Population data from the municipalities of hospital locations were obtained from the national censuses held in 2010 [40].

### Study variables

For the target variable we used the ratio of the number of applicants to the programme quota, according to a previous study [25], as an aggregated indicator of residents’ choice preferences.

Our main explanatory variable was the total amount of the resident’s salary throughout the 2-year residency programmes, based on Kawamura’s method [25]. Extra allowance for overtime work and night shift work was not included because it was likely to depend more on the resident’s duties rather than on monetary reward and the preferences of residents.

For comparative purposes, we adopted a set of covariates similar to those used in a previous study [25], such as the number of teaching physicians, hospital beds, emergency patients, and open/closed programmes within a single institution. The number of hospital beds should reflect the extent of hospital capacity and activity, which is further expected to parallel the volume and variety of inpatients with which the residents can gain experience during their training. However, because the number of hospital beds was under government regulation and was relatively constant over time, we replaced it in panel data analysis with the number of inpatients, which was more likely to vary over time. A closed programme within a single institution, compared with an open programme affiliated with other hospitals/clinics, was more likely to be provided by an institution with a wider variation of patients and more affluent educational resources, including teaching staff.

We further chose not to include several variables used in the previous study by Kawamura [25], such as the number of clinical departments and a dummy variable for the hospital’s location in metropolitan cities, because these features also do not change over time. Additionally, the number of departments was not reliable as an indicator of the size and function of hospitals because of non-standardized categorization of subspecialties under the current legal regulation of hospital administration in Japan [41]. Kawamura’s study [25] included the number of months for training of selective specialties in the programme as an index of programme attractiveness, but we did not include this information because it was specific to a programme rather than to a hospital.

### Statistical analysis

Descriptive statistics of hospital characteristics in 2-year periods were tested with the paired *t*-test. We conducted an OLS regression in the first step to replicate the results in Kawamura’s study as follows:

$$Y_{\mathrm{it}}=X_{\mathrm{it}}\beta +\mu_{\mathrm{it}}$$

where Y_*it*_ is the number of applicants to the quota of facility *i* in year *t*, X_*it*_ is a vector of explanatory variables including salary, and μ_*it*_ is the error term. We used data from 2009 for this analysis because they were more similar to the data from 2008 used for Kawamura’s analysis [25].

We further conducted panel data analysis with fixed and random effect models to eliminate the effect of unobserved heterogeneity of hospitals as follows:

$$Y_{\mathrm{it}}=X_{\mathrm{it}}\beta +D_{i}+\varepsilon_{\mathrm{it}}$$

where D_*i*_ is a vector reflecting time-consistent observed characteristics and unobserved heterogeneity of hospitals, and ε_*it*_ is the error term.

Because the urbanicity of hospital location would affect the impact of income, as well as hospital size and educational environments, we also performed stratified analysis by the population size of a city where the hospital was located. We divided hospitals into two groups by the median (that is, 279,127) of the population size. We also conducted ad hoc analysis with interaction terms as described in the Results section. We first obtained OLS estimation for comparative purposes with previous studies. We then additionally conducted panel data analyses with fixed and random effect models to evaluate misspecification bias. The estimated results are presented as elasticity for comparison purposes across the strata and models.

We conducted the *F*-test, Hausman test, and Breusch-Pagan test for model specification, or to identify the best model to address the effect of unobserved heterogeneity and within-hospital variance [42]. Data were analyzed with the statistical software STATA 11 (Stata Corporation, College Station, TX, USA).

## Results

Table 1 shows the descriptive statistics of the sample of hospitals included in the analysis. Over the studied period of 3 years, the salary of the 2-year residency was increased by 1.03 million yen ($ 12 875, *P* <0.001). The number of hospital beds (*P* <0.001), that of inpatients (*P* <0.001), and the proportion of closed training programmes (*P* <0.001) were significantly decreased on average over the same period. The number of teaching physicians and the average number of emergency patients were the same over this period.

**Table 1:** Description of statistical sample

	**2006**		**2009**		***P*-value**
**Variable**	**Mean**	**SD**	**Mean**	**SD**	
Ratio of the number of applicants to programme quota	0.71	0.68	0.68	0.59	0.24
Salary (million yen, per 2 years)	9.08	2.00	10.11	2.24	<0.001
Number of teaching physicians (persons per 100 beds)	10.4	3.1	10.4	3.7	0.92
Number of hospital beds	433	173	424	167	<0.001
Average number of inpatients (persons per day)	375	153	348	148	<0.001
Closed programme within a single institution (closed programme=1)	0.27	0.45	0.14	0.34	<0.001
Average number of emergency patients (persons per day per 10 beds)	0.92	0.58	0.92	0.96	0.91

Number of hospitals = 475.

Table 2 shows the results of regression models. The coefficients of variables included in the model were positive and statistically significant, but not for salary and the number of emergency patients. A larger number of teaching physicians, closed training programmes within a single institution, and a larger number of hospital beds were associated with a larger ratio of applicant number to quota.

**Table 2:** Multivariate analysis of the ratio of the number of applicants to programme quota by hospital characteristics

**Model**	**Ordinary least squares in 2009**	**Fixed effect**	**Random effect**
	Elasticity	Elasticity	Elasticity
Salary (million yen per 2 years)	0.0810	0.4995^*^	0.1480
Number of teaching physicians (persons per 100 beds)	0.6211^**^	0.3678^**^	0.6908^**^
Closed programme within a single institution (closed programme = 1)	0.0686^**^	−0.0096	0.0302^*^
Number of hospital beds (100 beds)	0.5154^**^		
Average number of emergency patients (persons per day per 10 beds)	0.0451	0.0309	0.0660^*^
Average number of inpatients (persons per day)		0.9316^**^	0.7145^**^

Number of hospitals = 475.

^*^P <0.05.

^**^P <0.01.

In the fixed effect model, salary showed a significant positive effect (ε = 0.4995, *P* = 0.015). The number of teaching physicians stayed significantly positive (ε = 0.3678, *P* = 0.004). The number of inpatients, replaced with that of hospital beds, also exhibited a positive effect (ε = 0.9316, *P* = 0.002) on the ratio of applicant number to quota. The number of emergency patients was positive (ε = 0.0309, *P* = 0.421) and the closed programme was negative (ε = −0.0096, *P* = 0.644), but these were not statistically significant.

In the random effect model, the results were similar to those of the pooled OLS, and salary was not statistically significant. The *F*-test of the model specification was highly significant (*P* <0.0001), in favor of the random effect model over the pooled OLS regression. The Breusch-Pagan test was also highly significant (*P* <0.0001), suggesting that the fixed model was better than the pooled OLS regression results. Finally, the Hausman test showed a *P*-value equal to 0.0001, suggesting that the results of the fixed effect model are the most relevant to address unobserved heterogeneity among hospitals.

Because the ratio in a smaller quota programme is more susceptible to a change in the number of applicants, we also conducted least squares regression weighted by hospital size, and first difference estimation with dummy variables for hospital size, and found significant coefficients of salary (data not shown).

Table 3 shows the results of analyses stratified by the city size of the hospital location. The elasticity of the salary in the OLS (ε = 0.5787, *P* = 0.027), the fixed effect model (ε = 0.6498, *P* = 0.036), and the random effect model (ε = 0.5470, *P* = 0.004), was positive and statistically significant in the small cities, but not significant in the large cities. When including an interaction term with the number of teaching physicians, however, the elasticity of salary became statistically significant and positive in the fixed effect model (ε = 1.9089, *P* = 0.016) and in the random effect model (ε = 1.7051, *P* = 0.004) in large cities, and the magnitude of the elasticity was comparable with that in small cities. The effect of the number of teaching physicians in the fixed effect model was positive and statistically significant, and larger in the large cities (ε = 1.9857, *P* = 0.009) compared with that in the small cities (ε = 1.6253, *P* = 0.033). Finally, the elasticity of the interaction term was negative and statistically significant in the fixed effect model (ε = −1.5223, *P* = 0.038) and in the random effect model (ε = −1.7013, *P* = 0.003) in large cities. The elasticity of the interaction term in small cities was also negative and marginally significant (ε = −1.4133, *P* = 0.052 for the fixed effect model; ε = −0.9690, *P* = 0.065 for the random effect model).

**Table 3:** Elasticity of the stratified analyses by the population size of the hospital location

**Model**		**Population <279,127**		**Population ≥279,127**	
		Elasticity	Elasticity	Elasticity	Elasticity
Ordinary least squares	Salary	0.5787^*^	1.0390	0.0278	1.3582
	Number of teaching physicians	0.5905^**^	1.0740	0.5724^**^	1.9154^*^
	Closed programme within a single institution	0.1052^**^	0.1042^**^	0.0537^**^	0.0543^**^
	Number of hospital beds	0.5751^**^	0.5707^**^	0.3919^**^	0.3821^**^
	Average number of emergency patients	0.1163	0.1182	0.0315	0.0319
	Interaction (Salary*Number of teaching physicians)		−0.5059		−1.3296
Fixed effects model	Salary	0.6498^*^	1.9185^**^	0.3842	1.9089^*^
	Number of teaching physicians	0.1985	1.6253^*^	0.4521^**^	1.9857^**^
	Closed programme within a single institution	0.0043	0.0056	−0.0152	−0.0114
	Number of inpatients	0.2155	0.1951	1.5433^**^	1.5960^**^
	Average number of emergency patients	0.0303	0.0053	0.0338	0.0370
	Interaction (Salary*Number of teaching physicians)		−1.4133		−1.5223^*^
Random effects model	Salary	0.5470^**^	1.4538^**^	0.0083	1.7051^**^
	Number of teaching physicians	0.5900^**^	1.5453^**^	0.6793^**^	2.3981^**^
	Closed programme within a single institution	0.0505^*^	0.0488^*^	0.0277	0.0307
	Number of inpatients	0.8792^**^	0.8692^**^	0.5482^**^	0.5413^**^
	Average number of emergency patients	0.1226	0.1231	0.0573	0.0585^*^
	Interaction (Salary*Number of teaching physicians)		−0.9690		−1.7013^**^
Number of hospitals		237	237	238	238

^*^P <0.05.

^**^P <0.01.

The results of the *F*-test, the Breusch-Pagan test, and the Hausman test in all four models suggested that the results of the fixed effects model were more relevant than those in the OLS and the random effect model.

## Discussion

We examined the effect of salary on residents’ hospital choices. The results of pooled OLS were similar to those of Kawamura’s previous results in that the coefficient of salary was positive and not significant [25]. However, the results of the fixed effect model were statistically significant, which were better supported by statistical specification tests. Basic characteristics of hospitals, such as hospital function, location, and ownership, are supposed to determine the attractiveness of hospitals and the level of income simultaneously [30,43]. Therefore, results that control for this unobserved heterogeneity and endogenous relationship between hospital characteristics and salary level should more precisely identify the effect of salary on residents’ choice of training hospitals.

Previous studies have also indicated that financial incentives would affect physicians’ choice of training hospitals or workplace in high-income countries as well as in middle-low income countries [13-15,27,28]. A systematic review on human resource allocation in healthcare reported that increased wage for trainees may be an effective strategy to improve supply as well as distribution of physicians, though the validity of evidence was limited due to poor study design [15]. With a sophisticated statistical technique and use of panel data, our results have added a strong support to the statement that monetary incentive is a significant factor for a physician’s choice of early career, especially in rural areas.

Our results also showed that the number of teaching physicians and that of inpatients had a positive effect on hospital choices. Previous studies also indicated that the quality of training environments, such as teaching skills of attending physicians and opportunities to learn clinical skills, was a significant factor that affected resident’s choice of specialty and training location, and job satisfaction [25,27,44,45]. Although the number of teaching physicians does not necessarily guarantee the quality of teaching, busy attending physicians were reported to decrease residents’ satisfaction with the quality of attending teaching in the US national survey of surgical residents [45]. The questionnaire survey by the MHLW in Japan also reported that in their choice of training, non-university residents took into consideration hospital factors, such as the number of clinical cases, to provide a sufficient learning experience (43.3%), the comprehensiveness of the training curriculum (37.5%), the number and quality of teaching physicians (29.7%), and high-tech therapeutic equipment of hospital facilities (27.3%) [27]. These conditions are expected to provide young residents with better opportunities to enhance their clinical skills and wider alternatives for career development.

In the current study in the analysis stratified by city size, the interaction term was statistically significant and negative in both groups, which suggested that the effects of salary and the number of teaching physicians were antagonistic. Therefore, the effect of salary was diminished when the number of teaching physicians was large. In addition, the magnitude of elasticity of the interaction and the number of teaching physicians were larger in large cities than those in small cities, suggesting that the antagonistic effect of the number of teaching physicians is larger in the urban setting. These results suggest that an increase in the number of teaching physicians may be a more effective strategy to attract residents in the urban setting than an increase in salary, while the increase in the number of teaching physicians may be a difficult alternative under the current shortage of physicians, especially in rural areas [46]. Offering a higher salary for young residents may be more feasible instead. Some previous studies argued that financial incentives alone are not sufficient, and that the strategy for staffing remote rural areas should be multi-facetted and comprehensive [6,8,13]. Our results rather indicated that resource could be strategically allocated between monetary incentives and training environments, according to local conditions.

Our study has several limitations. First, almost half of the hospitals included in the national residency programme were excluded from our sample because of the limitation in data availability. Because the submission of hospital information was voluntary, hospitals with a small-scale programme tended to be excluded from the Guidebook. We compared all the hospitals included in the Guidebook (n = 705 in 2006, and 683 in 2009) with those included in our panel analysis. We found that the number of teaching physicians in 2006 was smaller in all the hospitals included in the Guidebook than those included in our panel analysis (10.11 persons versus 10.40 persons, *P* = 0.04), and the number of inpatients was also smaller in 2006 and 2009 (in 2006, 359 versus 375 patients, *P* = 0.03; in 2009, 332 versus 348 patients, *P* = 0.02). Otherwise, the characteristics were comparable between the two datasets. In our study, the descriptive statistics on the number of hospital beds or doctors were similar to those of Kawamura’s study, which had a cross-sectional and larger sample than that in our study [25]. However, in 2009 in the university-affiliated hospitals, the average number of beds was 596, the average number of outpatients was 977 per day, and the average number of doctors was 47 per 100 hospital beds [47]; these numbers were much larger than in our sample. Therefore, we acknowledge that our estimation of the effect of salary in non-university hospitals may not necessarily hold true in the university hospitals. We also cannot deny the possibility that our results may have limited generalizability, and these should be confirmed with longer-term and larger panel data from teaching hospitals in the country.

Second, the natural change in residents’ preferences over the studied period could have affected our results. For example, the number of female physicians has been continuously increasing to more than 30% of newly qualified physicians [48]. Sex is significantly related to a resident’s choice of a university hospital as a training site [28], and also with the likelihood of satisfaction with salary [49]. Because the number of residents by sex was not available, we could not account for the effect of the resident’s sex in our analysis. The results of this study should be confirmed with sex-specific datasets, or more preferably, with microdata of residents’ preferences.

## Conclusions

Our results show that an increase in salary has a positive and significant effect on a resident’s choice of training hospital among non-university institutions. The conditions of monetary reward in a residency programme are a possible tactic for hospitals to attract residents.

## Abbreviations

JRMP: Japan Residency Matching Programme; MHLW: Ministry of Health, Labour, and Welfare; OLS: ordinary least squares; PMEP: Postgraduate Medical Education Programme.

## Competing interests

The authors declare that they have no competing interests.

## Authors’ contributions

TE designed the study, collected the data, and analyzed and interpreted the data. TE wrote the first draft of the manuscript. HH supervised the study and critically reviewed the manuscript. TE and HH read and approved the final manuscript.
